# Complete plastome sequence of *Euphorbia milii* Des Moul. (Euphorbiaceae)

**DOI:** 10.1080/23802359.2019.1703598

**Published:** 2020-01-08

**Authors:** Yi-Li Jiang, Hong-Xin Wang, Zhi-Xin Zhu, Hua-Feng Wang

**Affiliations:** Key Laboratory of Tropical Biological Resources of Ministry of Education, School of Life and Pharmaceutical Sciences, Hainan University, Haikou, China

**Keywords:** *Euphorbia milii*, plastome, phylogeny, genome structure, Euphorbiaceae

## Abstract

*Euphorbia milii* (Euphorbiaceae) grows as a scrambling shrub with many branches. Here, we report and characterize the complete plastome of *E. milii* in an effort to provide genomic resources useful for promoting its systematic research. The plastome of *E. milii i*s found to possess a total length of 160,806 bp with the typical quadripartite structure of angiosperms, contains two Inverted Repeats (IRs) of 26,695 bp, a Large Single-Copy (LSC) region of 90,211 bp and a Small Single-Copy (SSC) region of 17,205 bp. The plastome contains 114 genes, consisting of 80 unique protein-coding genes, 30 unique tRNA genes and four unique rRNA genes. The overall A/T content in the plastome of *E. milii* is of 64.10%. The phylogenetic analysis indicated that *E. milii* is close to *E. tirucalli* within Euphorbiaceae in this study. The complete plastome sequence of *E. milii* will provide a useful resource for the conservation genetics of this species as well as for the phylogenetic studies of Euphorbiaceae.

## Introduction

*Euphorbia milii* Des Moul. (Euphorbiaceae) grows as a scrambling shrub with many branches. It easily reaches height of 60–90 cm. It is native to Africa (Madagascar) and widely cultivated as an ornamental plant in the tropical and temperate regions of the old continent. It could be of medicinal use (Ma and Gilbert 2008). Consequently, the genetic and genomic information is needed to promote its systematic research and further development of cultivation value of *E. milii.* Here, we report and characterize the complete plastome of *E. milii* (GenBank accession number: MN713924). This is the first report of a complete plastome for *E. milii.*

In this study, *E. milii* was sampled from the greenhouse within Hainan University campus, Haikou, Hainan, China (110.327°E, 20.059°N). A voucher specimen (Wang et al., B257) and its DNA was deposited in the Herbarium of the Institute of Tropical Agriculture and Forestry (HUTB), Hainan University, Haikou, China.

The experiment procedure is as reported in Zhu et al. (2018). Around six Gb clean data were assembled against the plastome of *E. esula* (NC_033910.1) (Horvath et al. 2017) using MITObim v1.8 (Hahn et al. 2013). The plastome was annotated using Geneious R8.0.2 (Biomatters Ltd., Auckland, New Zealand) against the plastome of *E. esula* (NC_033910.1). The annotation was corrected with DOGMA (Wyman et al. 2004).

The plastome of *E. milii* is found to possess a total length 160,806 bp with the typical quadripartite structure of angiosperms, contains two Inverted Repeats (IRs) of 26,695 bp, a Large Single-Copy (LSC) region of 90,211 bp and a Small Single-Copy (SSC) region of 17,205 bp. The plastome contains 114 genes, consisting of 80 unique protein-coding genes, 30 unique tRNA genes and four unique rRNA genes. The overall A/T content in the plastome of *E. milii* is 64.10%, which the corresponding value of the LSC, SSC, and IR region were 66.80%, 69.40%, and 57.70%, respectively.

**Figure 1.**
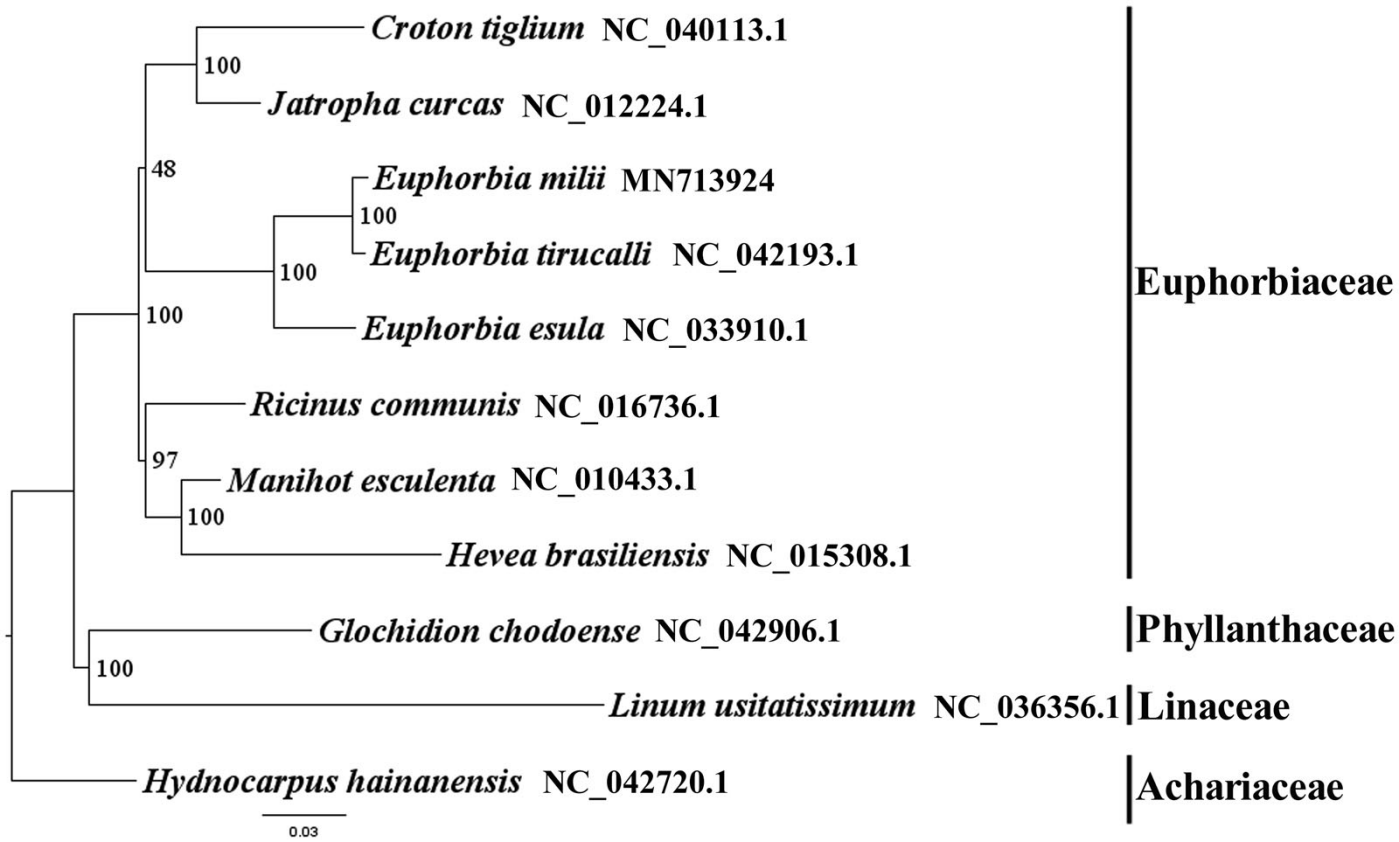
The best maximum likelihood (ML) phylogeny recovered from 11 complete plastome sequences by RAxML. Accession numbers: *Euphorbia milii* MN713924, *Euphorbia tirucalli* NC_042193.1, *Euphorbia esula* NC_033910.1, *Croton tiglium* NC_040113.1, *Jatropha curcas* NC_012224.1, *Ricinus communis* NC_016736.1, *Manihot esculenta* NC_010433.1, *Hevea brasiliensis* NC_015308.1, *Glochidion chodoense* NC_042906.1, *Linum usitatissimum* NC_036356.1, *Hydnocarpus hainanensis* NC_042720.1.

We used RAxML (Stamatakis 2006) with 1,000 bootstraps under the GTRGAMMAI substitution model to reconstruct a maximum likelihood (ML) phylogeny of seven published complete plastomes of Euphorbiaceae, using three species of three families (Phyllanthaceae, Linaceae, Achariaceae) as outgroups. The phylogenetic analysis indicated that *E. milii* is close to *E. tirucalli* within Euphorbiaceae in this study (Figure 1). Most nodes in the plastome ML tree were strongly supported. The complete plastome sequence of *E. milii* will provide a useful resource for the conservation genetics of this species as well as for the phylogenetic studies of Euphorbiaceae.
